# *N*-Eicosapentaenoyl Dopamine, A Conjugate of Dopamine and Eicosapentaenoic Acid (EPA), Exerts Anti-inflammatory Properties in Mouse and Human Macrophages

**DOI:** 10.3390/nu11092247

**Published:** 2019-09-18

**Authors:** Giuseppina Augimeri, Pierluigi Plastina, Giulia Gionfriddo, Daniela Rovito, Cinzia Giordano, Alessia Fazio, Ines Barone, Stefania Catalano, Sebastiano Andò, Daniela Bonofiglio, Jocelijn Meijerink, Renger Witkamp

**Affiliations:** 1 Department of Pharmacy, Health and Nutritional Sciences, University of Calabria, 87036 Arcavacata di Rende (CS), Italy; giusy.augimeri@gmail.com (G.A.); giu.gionfriddo@gmail.com (G.G.); rovitod@igbmc.fr (D.R.); cinzia.giordano@unical.it (C.G.); alessia.fazio@unical.it (A.F.); ines.barone@unical.it (I.B.); stefania.catalano@unical.it (S.C.); sebastiano.ando@unical.it (S.A.); 2 Division of Human Nutrition and Health, Wageningen University, 6700 AA Wageningen, The Netherlands; jocelijn.meijerink@wur.nl

**Keywords:** cyclooxygenase-2, cytokines, endocannabinoid, inflammation, *N*-acyl dopamine, *N*-eicosapentaenoyl dopamine, polyunsaturated fatty acids (PUFAs)

## Abstract

A large body of evidence suggests that dietary *n*-3 polyunsaturated fatty acids (PUFAs), including eicosapentaenoic acid (EPA) and docosahexaenoic acid (DHA), contribute to a reduced inflammatory tone thereby lowering the risk for several chronic and degenerative diseases. Different mechanisms have been proposed to explain these anti-inflammatory effects, including those involving endocannabinoids and endocannabinoid-like molecules. In this context, fatty acid amides (FAAs), conjugates of fatty acids with amines or amino acids, are an emerging class of compounds. Dopamine conjugates of DHA (*N*-docosahexaenoyl dopamine, DHDA) and EPA (*N*-eicosapentaenoyl dopamine, EPDA) have previously been shown to induce autophagy, apoptosis, and cell death in different tumor lines. Additionally, DHDA has displayed anti-inflammatory properties in vitro. Here, we tested the immune-modulatory properties of EPDA in mouse RAW 264.7 and human THP-1 macrophages stimulated with lipopolysaccharide (LPS). EPDA suppressed the production of monocyte chemoattractant protein-1 (MCP-1), and interleukin-6 (IL-6) in both cell lines, and nitric oxide (NO), and macrophage-inflammatory protein-3α (MIP3A) in RAW 264.7 macrophages. At a transcriptional level, EPDA attenuated cyclooxygenase-2 (COX-2) expression in both cell lines and that of MCP-1, IL-6, and interleukin-1β (IL-1β) in THP-1 macrophages. Although further research is needed to reveal whether EPDA is an endogenous metabolite, our data suggest that this EPA-derived conjugate possesses interesting immune-modulating properties.

## 1. Introduction

Eicosapentaenoic acid (EPA; 20:5(*n*-3)) and docosahexaenoic acid (DHA; 22:6(*n*-3)) are long-chain omega-3 polyunsaturated fatty acids (*n*-3 LC-PUFAs) found in significant amounts in seafood, particularly in oily fish. Although EPA and DHA can be biosynthesized from plant-derived α-linolenic acid (ALA), this metabolic pathway is not very efficient in adults. Omega-3 PUFAs represent key molecules for normal tissue development, and contribute to the prevention and/or management of certain cardiovascular, neurological, and other diseases [1,2]. The positive effects of *n*-3 LC-PUFAs are assumed to be linked to their ability to mitigate chronic low-grade inflammatory processes in various type of cells, including macrophages. These immune cells represent the key players in the initial phases of an inflammatory reaction, which can resolve or develop into chronic inflammation. The latter condition presents risk factors for the above-mentioned disorders [3,4,5,6]. To explain the anti-inflammatory activity of *n*-3 LC-PUFAs, different mechanisms have been suggested which are at least partly connected and acting in parallel [5,6]. EPA and DHA have been found to directly interact with receptors and key regulators of inflammation [7,8]. It has been demonstrated that *n*-3 LC-PUFAs can bind to the G protein-coupled receptor GPR120 [9], as well as peroxisome proliferator-activated receptors (PPARs) [10]. Alternatively, indirect mechanisms have been suggested, involving the incorporation of *n*-3 PUFAs in cell membrane phospholipids instead of the more abundant *n*-6 arachidonic acid (AA). This eventually leads to a shift from the production of AA-derived prostaglandins (PGs) and leukotrienes (LTs) towards less potent pro-inflammatory or even anti-inflammatory pathways [5,6]. In addition, EPA and DHA can be transformed into resolvins and protectins, which are considered to mostly regulate the extent and magnitude of inflammatory processes [11].

**Figure 1:**
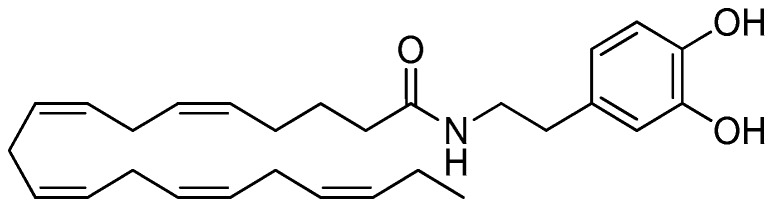
*N*-Eicosapentaenoyl dopamine (EPDA).

Over the last decade, we and others have proposed that fatty acid amides (FAAs) derived from *n*-3 PUFAs can account at least partly for their anti-inflammatory activities [12,13,14]. FAAs are conjugates of fatty acids with amines, including ethanolamine, dopamine and serotonin, or amino acids. These compounds are widely diffused in nature and display a number of bioactivities [15]. *n*-3 PUFA-derived FAAs represent a relatively unknown sub-class with specific properties which are only quite recently receiving attention [12,13,14]. Among them, *N*-docosahexaenoyl serotonin (DHA-5-HT) reduces the production of pro-inflammatory markers in macrophages and human peripheral blood mononuclear cells [16,17]. More recently, *N*-eicosapentaenoyl vanillylamine (EPVA) and *N*-docosahexaenoyl vanillylamine (DHVA) were shown to possess anti-inflammatory properties in mouse macrophages [18]. Other *n*-3 PUFA-derived FAAs, namely *N*-eicosapentaenoyl ethanolamine (EPEA) and *N*-docosahexaenoyl ethanolamine (DHEA) have been reported to exert anti-inflammatory properties in adipocytes and macrophages [19,20]. In particular, a diminished production of cyclooxygenase 2 (COX-2)-mediated eicosanoids has been shown to mainly contribute to the anti-inflammatory actions of DHEA in macrophages, while the involvement of cannabinoid receptors has been ruled out [21]. More recently, the preferential activation of CB2 cannabinoid receptors by *n*−3 PUFA ethanolamides has been suggested to contribute to other processes in immune cells, possibly migration [22]. Interestingly, both ethanolamides were also found to exert cytotoxic activity in prostate and breast cancer cell lines [23,24]. Similarly, *N*-docosahexaenoyl dopamine (DHDA) and *N*-eicosapentaenoyl dopamine (EPDA), which are putative metabolites of DHA and EPA, respectively, have been reported to display antitumor activity in breast cancer cells [25], whereas DHDA was found to exert anti-inflammatory properties in macrophages and microglial cells [26,27]. Remarkably, Dang and coworkers demonstrated that compounds bearing a dopamine head group were the most active in reducing pro-inflammatory markers among a series of structural analogues [26]. However, EPDA has not been studied in this respect. Therefore, the anti-inflammatory potential of EPDA (Figure 1) was investigated in the present study using mouse macrophages. Moreover, the anti-inflammatory potential of EPDA was also explored in a cell line of human origin, to verify the spectrum of its immune-modulating properties. 

## 2. Materials and Methods 

### 2.1. Chemicals and Materials

Eicosapentaenoic acid, Griess reagents, and nitrite standard were obtained from Cayman Chemical (Ann Arbor, MI, USA). Novozym®435 (consisting of immobilized *Candida antarctica* Lipase B) was supplied by Novozymes A/S (Bagsværd, Denmark). Lipopolysaccharide (O111:B4; LPS), dopamine hydrochloride, 2-methyl-2-butanol, triethylamine, phorbol 12-myristate 13-acetate (PMA), 3-(4,5-dimethylthiazol-2-yl)-2,5-diphenyltetrazolium (MTT), dimethyl sulphoxide (DMSO), glutamine, HEPES and 2-mercaptoethanol were from Sigma-Aldrich (Schnelldorf, Germany). Dulbecco’s modified Eagle’s medium (DMEM), Roswell Park Memorial Institute 1640 (RPMI-1640) medium, penicillin, streptomycin, and fetal bovine serum (FBS) were purchased from Lonza (Verviers, Belgium). ELISA kits for interleukin-6 (IL-6), macrophage-inflammatory protein-3α (MIP3A), inflammatory chemokine monocyte chemotactic protein-1 (MCP-1), were purchased from R&D Systems (Abingdon, UK).

### 2.2. One-Step Enzymatic Synthesis of N-Eicosapentaenoyl Dopamine

*N*-Eicosapentaenoyl dopamine was synthesized using an enzymatic *N*-acylation method previously developed by our team [27,28]. Briefly, EPA (121 mg, 0.4 mmol) was let to react with dopamine hydrochloride (76 mg, 0.4 mmol), in the presence of Novozym®435 as the catalyst (100 mg), an excess of triethylamine (60 mg, 0.6 mmol), in 2-methyl-2-butanol (2 mL), in an orbital shaker at 50°C for 48 h. After cooling, evaporation of the solvent under reduced pressure and filtration of the enzyme, column chromatography on silica gel (*n*-hexane-acetone was used as the eluent) gave EPDA (yield 98 mg, 56%). The product was characterized by ESI-MS, ^1^H NMR, ^13^C NMR, and IR. Characterization data are collected in Figure S1.

### 2.3. Cell Culture

RAW264.7 mouse macrophages (American Type Culture Collection (ATCC), Teddington, UK) were maintained in DMEM added with FBS (10%), streptomycin and penicillin (1%), at 37°C under a humidified atmosphere (5% CO_2_/95% air), in 75 cm^2^ cell culture flasks (Corning, Acton, MA, USA) and passaged twice a week. Cells were seeded into 96-well plates (250,000 cells mL^−1^) for viability and cytotoxicity assays, 48-well plates (250,000 cells mL^−1^) for nitrite assay and ELISAs, or 6-well plates (500,000 cells mL^−1^) for RNA extraction. After overnight incubation, attached RAW264.7 cells were exposed to 0.5 µg mL^−1^ LPS (or not, untreated cells) to induce an inflammatory response, with (or without) EPDA (concentration range 0.01–2.5 μM) for 24–48 h, depending on the experimental set-up. The human monocyte THP-1 cell line (TIB-202) was purchased from ATCC (Manassas, VA, USA) and cultured in RPMI-1640 medium supplemented with 10% FBS, 1% glutamine, 1% HEPES and 2-mercaptoethanol (to a final concentration of 0.05 mM) at 37°C in a humidified atmosphere (5% CO_2_/95% air). THP-1 cells were seeded in 6-well plates at a density of 1,000,000 cells/well (for RNA extraction) or in 24-well plates at a density of 100,000 cells/well (for MTT assay and ELISAs) in the presence of PMA (100 nM) to let differentiation into macrophages. Following overnight incubation and replacing of PMA-containing medium with fresh medium, macrophages were let to rest for 24 h and then exposed to 10 ng mL^−1^ LPS (or not, untreated cells) with (or without) EPDA (1 μM) for 4 h (for RNA extraction) or 24 h for ELISAs. In all cases, absolute ethanol was used to dissolve test compounds and these solutions were diluted 1:1000 in culture medium. The final solvent concentration did not exceed 0.1% (*v*/*v*) in the culture medium.

### 2.4. Viability and Cytotoxicity Assays

Viability of RAW264.7 cells was evaluated by determining tetrazolium salt (XTT) conversion using an XTT Cell Proliferation Kit II (Roche Applied Science, Almere, The Netherlands), while potential cytotoxicity effects of EPDA were evaluated by measuring lactate dehydrogenase (LDH) leakage using a Cytotoxicity Detection Kit (Roche Applied Science, Almere, The Netherlands), as previously reported [18]. Briefly, after incubating RAW264.7 cells with (or without) EPDA (concentration range 0.01–2.5 μM) and LPS (0.5 µg mL^−1^) for 48 h, supernatants were removed and used for LDH determination. Next, fresh medium (100 µL) containing sodium 30-[1-(phenylaminocarbonyl)-3,4-tetrazolium]bis(4-methoxy-6-nitro) benzenesulfonic acid hydrate (XTT) (final concentration = 0.45 mM) and *N*-methyldibenzopyrazine methylsulfate (1.25 mM), was added to the cells. After incubating for 30 min at 37°C, the quantity of formazan formed in the medium was evaluated at 450 nm on a plate reader (Multiskan Ascent, ThermoLabsystem, Breda, The Netherlands). LDH was analyzed in culture supernatants (100 µL), previously taken that were added with enzyme reagents (diaphorase/NAD mixture, 250 µL) and dye solutions (iodotetrazolium chloride and sodium lactate, 11.25 mL). After incubating for 30 min at 25 °C, the absorbance was measured at 492 nm. Cell viability in THP-1 macrophages was determined by using the 3-(4,5-dimethylthiazol-2-yl)-2,5-diphenyltetrazolium (MTT) assay. THP-1 cells were differentiated in 24-well plates (100,000 cells/well) as previously described and exposed to LPS (10 ng mL^−1^) with (or without) EPDA for 24, 48, and 72 h. MTT (2 mg mL^−1^) was added to each well, and the plates were incubated for 2 h at 37 °C. After the removal of the medium and the addition of DMSO (500 μL), absorbance was measured at 570 nm.

### 2.5. Determination of Nitric Oxide Production by Griess Assay

RAW 264.7 cells were plated at a density of 250,000 cells mL^−1^ in 48-well plates, and incubated overnight. Adherent cells were exposed to 0.5 μg mL^−1^ LPS (or not, untreated cells) with (or without) EPDA (concentration range 0.01–2.5 μM), and then incubated for 48 h. The amount of nitrite, used as an estimation for nitric oxide (NO) production, was measured as described previously [18]. Briefly, supernatants (100 μL) were mixed with Griess reagent (100 μL), according to manufacturer’s instructions. The mixture was incubated for 10 min at room temperature and the absorbance at 540 nm was measured using an ELISA plate reader. A calibration curve with sodium nitrite as the standard was used for quantification. 

### 2.6. Determination of IL-6, MCP-1, and MIP3A Production by ELISA

RAW 264.7 cells were seeded at a density of 250,000 cells mL^−1^ in 48-well plates and incubated overnight. Adherent cells were exposed to 0.5 μg mL^−1^ LPS (or not, untreated cells) with (or without) EPDA (concentration range 0.01–2.5 μM). After 24 h incubation, the supernatants were used for measuring of MIP3A, MCP-1, and IL-6 levels by mouse ELISA kits according to the manufacturer’s instructions. THP-1 cells were differentiated as previously described in 24-well plates (100,000 cells/well) and exposed to 10 ng mL^−1^ LPS (or not, untreated cells) with (or without) EPDA (1.0 μM) for 24 h. Then, the supernatants were used for measuring of MCP-1 and IL-6 levels by human ELISA kits according to the manufacturer’s instructions. 

### 2.7. RNA Purification and Quantitative Reverse Transcription Real-Time PCR

Total RNA was extracted using TRIzol reagent (Invitrogen, Breda, The Netherlands). RNA (1 μg/sample for RAW264.7 or 2 μg/sample for THP-1 cells) was reverse transcribed to give complementary DNA (cDNA) using the reverse transcription system from Promega (Leiden, The Netherlands) or Applied Biosystems (Monza, Italy) for RAW264.7 or THP-1 cells, respectively. cDNA was amplified by PCR using the master-mix Sensimix SYBR (Bioline Reagents Ltd., London, UK) on a CFX Real Time system apparatus (Bio-Rad, Veenendaal, The Netherlands) or by using the master-mix SYBR Green (Biorad, Hercules, CA, USA) on a iCycler iQ Detection System (Bio-Rad, Milan, Italy), for RAW264.7 or THP-1 cells, respectively. The following primer pairs were used for amplification of mouse COX-2: 5′-TGAGCA-ACT-ATT-CCA-AAC-CAG-C-3′ (forward) and 5′-GCA-CGTAGT-CTT-CGA-TCA-CTA-TC-3′ (reverse). Samples were analyzed in duplicate, and mRNA levels of the COX-2 gene were normalized to RPS27A2. Primer pairs for RPS27A2 were 5′-GGT-TGA-ACCCTC-GGA-CAC-TA-3′ (forward) and 5′-GCC-ATC-TTC-CAGCTG-CTT-AC-3′ (reverse). The following primer pairs were used for amplification of human COX-2: 5′-TAA-GTG-CGA-TTG-TAC-CCG-GAC -3′ (forward) and 5′-TTT-GTA-GCC-ATA-GTC-AGC-ATT-GT -3′ (reverse), IL-1β: 5′-TGA-TGG-CTT-ATT-ACA-GTG-GCA-ATG-3′ (forward) and 5′-GTA-GTG-GTG-GTC-GGA-GAT-TCG (reverse), IL-6: 5′-CCA-GGA-GCC-CAG-CTA-TGA-AC-3′ (forward) and 5′-CCC-AGG-GAG-AAG-GCA-ACT-G-3′ (reverse), MCP-1: 5′-CAG-CCA-GAT-GCA-ATC-AAT-GCC-3′ (forward) and 5′-TGG-AAT-CCT-GAA-CCC-ACT-TCT-3′. Samples were analyzed in duplicate, and mRNA levels of the different genes were normalized to 18S. Primer pairs for 18S were 5′-CGG-CGA-CGA-CCC-ATT-CGA-AC -3′ (forward) and 5′-GAA-TCG-AAC-CCT-GAT-TCC-CCG-TC -3′ (reverse). 

### 2.8. Statistical Analysis

At least three independent experiments performed on different days were carried out in duplicate or triplicate. Data from all experiments are expressed as percentages of the LPS-treated controls (set at 100%) and presented as means ± standard deviation (SD). Statistical differences between treatments and controls were evaluated by one-way ANOVA followed by Dunnett’s post hoc test. *p* values <0.05 (*), and *p* < 0.01 (**) were considered as statistically significant. 

## 3. Results

### 3.1. EPDA is not Cytotoxic at the Concentrations Tested

Possible cytotoxic effects of EPDA were considered by carrying out XTT and LDH assays in RAW264.7 cells (EPDA concentration range of 0.01–2.5 μM) and MTT assay in THP-1 macrophages (EPDA concentration range of 0.01–1.0 μM). Cell proliferation and cytotoxicity were found not to differ more than 20% with respect to vehicle control. Therefore, these not cytotoxic conditions were used for further experiments. Data are reported in Table S1 and Table S2 (Supporting Information).

### 3.2. EPDA Reduces Secreted NO, IL-6, MCP-1, and MIP3A in LPS-Stimulated RAW 264.7 Macrophages

**Figure 2:**
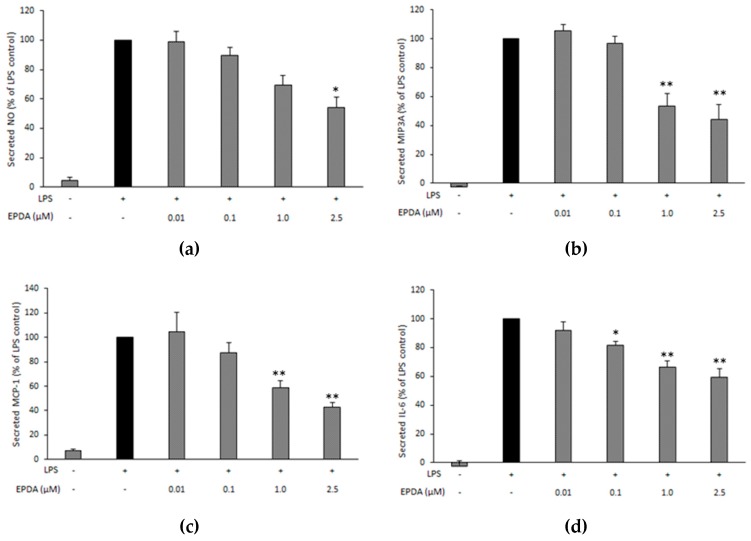
Effect of *N*-eicosapentaenoyl dopamine (EPDA) on the secreted nitric oxide (NO) (**a**), macrophage-inflammatory protein-3α (MIP3A) (**b**), chemokine monocyte chemoattractant protein-1 (MCP-1) (**c**) and interleukin-6 (IL6) (**d**) by RAW264.7 macrophages exposed to lipopolysaccharide (LPS). Cells were seeded in 48-well plates at 250,000 cells mL^−1^. After overnight incubation, cells were treated with (or without) LPS (0.5 μg mL^−1^) and with (or without) EPDA (concentration range of 0.01–2.5 μM) for 24 h (for proteins) or for 48h (for NO). Cell supernatants were evaluated for nitrite concentration and secreted proteins using an ELISA reader. Results are expressed as percentages of LPS control (black bar, set at 100%), and represent the mean of three independent experiments (each done in duplicate) ± S.D. Average absolute value for nitrite production with LPS stimulation (LPS control) after 48 h was 24 ± 5 µM. Average absolute values for MIP3A, MCP-1, and IL-6 with LPS stimulation (LPS control) after 24 h were 384 ± 67 pg mL^−1^, 38 ± 3 ng mL^−1^, 74 ± 4 ng mL^−1^, respectively. Asterisks indicate significantly different mean values from those of control (* *p* < 0.05, and ** *p* < 0.01).

To explore the potential anti-inflammatory effects of EPDA, RAW264.7 cells were stimulated by 0.5 μg mL^−1^ LPS (or not, untreated cells, - LPS) with (or without, containing vehicle only) EPDA in a concentration range of 0.01–2.5 μM. Cells were incubated for 48 h, as NO is a late mediator in the LPS-stimulated inflammatory pathway [29]. EPDA decreased secreted NO at the highest concentration (2.5 μM) by 46% compared to the LPS-treated control (Figure 2a). 

We further investigated the potential of EPDA on three pro-inflammatory mediators, namely MIP3A, MCP-1, and IL-6. Concentration−dependent effects of EPDA on the secreted markers were observed (Figure 2b–d). After 24 h incubation, 2.5 μM EPDA significantly inhibited the release of MIP3A (Figure 2b), MCP-1 (Figure 2c), and IL-6 (Figure 2d) by 56%, 57%, and 41%, respectively, compared to LPS-treated control. Remarkably, EPDA was able to significantly suppress IL-6 release also at 0.1 μM.

### 3.3. EPDA Reduces COX-2 Expression in LPS-Stimulated RAW264.7 Macrophages

**Figure 3:**
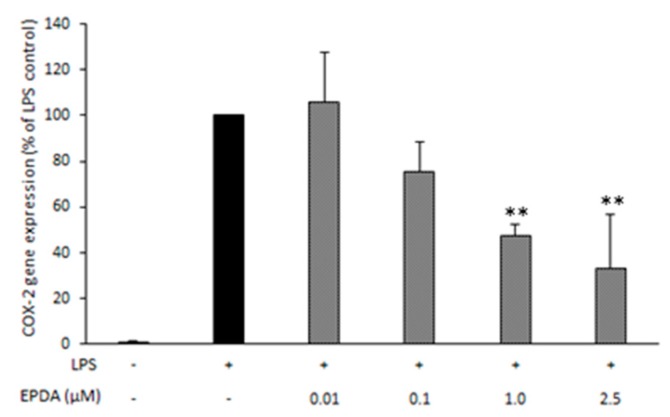
Effect of *N*-eicosapentaenoyl dopamine (EPDA) on cyclooxygenase-2 (COX-2) gene expression by RAW264.7 macrophages exposed to lipopolysaccharide (LPS). Cells were seeded in 6-well plates at 500,000 cells mL^−1^. After overnight incubation, cells were treated with (or without) LPS (0.5 μg mL^−1^) and with (or without) EPDA (concentration range of 0.01–2.5 μM) for 24 h. Each sample was normalized on its RPS27A2 mRNA content. Data are expressed as percentages of LPS control (black bar, set at 100%), and represent the mean of three independent experiments (each done in duplicate) ± S.D. Asterisks indicate different mean values from those of control (** *p* < 0.01).

In order to investigate possible involvement of COX-2-mediated processes in the apparent anti-inflammatory pathways observed, we used quantitative RT-PCR to determine COX-2 mRNA expression in RAW264.7 macrophages. A concentration−dependent decrease of COX-2 gene expression was observed, with a maximum reduction of 67% compared to LPS-treated control at a concentration of 2.5 μM of EPDA (Figure 3).

### 3.4. EPDA Reduces Secreted MCP-1, and IL-6 in LPS-Stimulated THP-1 Macrophages

**Figure 4:**
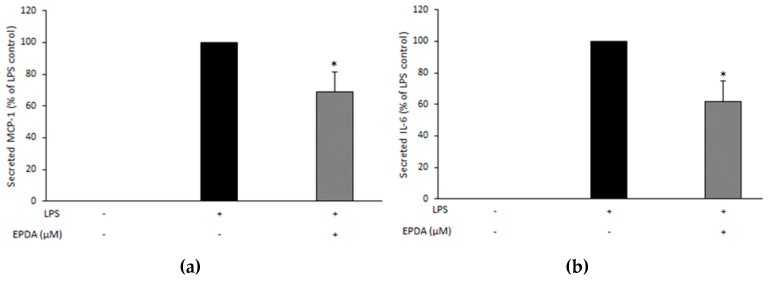
Effect of *N*-eicosapentaenoyl dopamine (EPDA) on the secreted chemokine monocyte chemoattractant protein-1 (MCP-1) (**a**) and interleukin-6 (IL6) (**b**) by THP-1 macrophages exposed to lipopolysaccharide (LPS). Cells were seeded in 24-well plates at 100,000 cells/well in the presence of PMA (100 nM). After overnight incubation, and replacing of PMA-containing medium with fresh medium, attached cells were rested for 24 h and then stimulated with (or without) LPS (10 ng mL^−1^) and with (or without) EPDA (1 μM) for 24 h. Cell supernatants were evaluated for secreted proteins using ELISA. Data are expressed as percentages of LPS control (black bar, set at 100%), and represent the mean of three independent experiments (each done in triplicate) ± SD. Average absolute values for MCP-1 and IL-6 with LPS stimulation (LPS control) after 24 h were 86 ± 5 and 213 ± 26 pg mL^−1^, respectively. Asterisks indicate significantly different mean values from those of control (* *p* < 0.05).

In order to compare our findings in murine RAW 264.7 cells with those in a human cell line, we exposed PMA-differentiated THP-1 cells stimulated with (and without) LPS (10 ng mL^−1^) to EPDA (1 μM) and measured the effects on MCP-1 and IL-6 production. As it can be seen in Figure 4, EPDA significantly reduced the secreted pro-inflammatory markers, by 31% and 38%, respectively, compared to LPS-treated control.

### 3.5. EPDA Reduces MCP-1, IL-6, COX-2, and IL-1β Gene Expression in LPS-Stimulated THP-1 Macrophages

**Figure 5:**
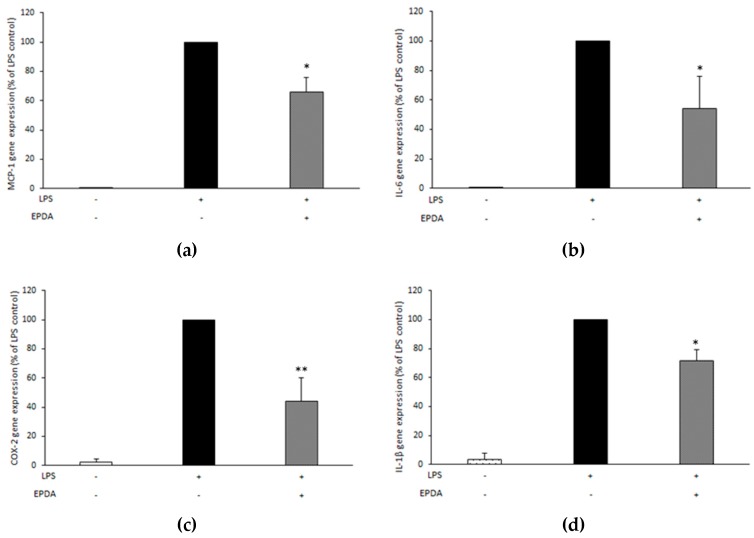
Effect of *N*-eicosapentaenoyl dopamine (EPDA) on the gene expression of MCP-1 (**a**), IL-6 (**b**), cyclooxygenase-2 (COX-2) (**c**), and interleukin-1β (IL-1β) (**d**) by THP-1 macrophages exposed to lipopolysaccharide (LPS). Cells were seeded in 6-well plates at 1,000,000 cells/well in the presence of PMA (100 nM). After overnight incubation, and replacing of PMA-containing medium with fresh medium, attached cells were rested for 24 h and then stimulated with (or without) LPS (10 ng mL^−1^) and with (or without) EPDA (1 μM) for 4 h. Each sample was normalized on its 18S mRNA content. Data are expressed as percentages of LPS control (black bar, set at 100%), and represent the mean of three independent experiments (each done in triplicate) ± S.D. Asterisks indicate significantly different mean values from those of control (* *p* < 0.05, and ** *p* < 0.01).

In order to investigate whether the effects of EPDA on MCP-1 and IL-6 were also paralleled by changes at the gene expression level in LPS-stimulated THP-1 macrophages, we used quantitative RT-PCR to determine MCP-1, IL-6, COX-2, as well as IL-1β mRNA expression. A significant reduction (by 34%, 46%, 56%, and 28%, respectively, compared to LPS-treated control) was observed for these genes upon treatment with EPDA at a concentration of 1.0 μM (Figure 5).

## 4. Discussion

Results from previous studies by our team and other groups consistently show that *n*-3 LC PUFA-derived amides possess more potent anti-inflammatory properties than their analogues bearing shorter or saturated chains [17,18,20,26]. The serotonin conjugate of DHA (DHA-5-HT) was identified as the most potent inhibitor of interleukin-17 (IL-17) and MIP3A secreted by blood mononuclear cells among a series of *N*-acyl serotonins [17]. DHA-5-HT was also found to attenuate the IL-23-IL-17 signaling axis in LPS-stimulated RAW264.7 macrophages [16]. The levels of NO, prostaglandin 2 (PGE_2_), IL-6, IL-1β, and interleukin-23 (IL-23) were reduced by DHA-5-HT at sub-micromolar concentration (0.1–0.5 µM). Moreover, DHA-5-HT inhibited the ability of RAW264.7 cells to migrate and reduced chemokines such as MCP-1 and MIP3A [16]. The *n*-3 PUFA-derived vanillylamides EPVA and DHVA were shown to reduce some LPS-induced inflammatory mediators, including NO, MCP-1, and MIP3A by RAW264.7 macrophages [18]. The ethanolamide of DHA (DHEA) was found as the most potent inhibitor of NO release by LPS-induced RAW264.7 macrophages from a series of *N*-acyl ethanolamines (NAEs) with different chain length and degree of unsaturation [20]. Moreover, DHEA suppressed MCP-1 release in the same cell line. Inhibitory properties of DHEA were also found in LPS-stimulated mouse peritoneal macrophages, as DHEA reduced MCP-1 and NO levels [20]. DHEA and its congener EPEA have been reported to reduce LPS-induced MCP-1 and IL-6 levels in 3T3-L1 adipocytes [19]. Interestingly, Dang and coworkers demonstrated that FAAs bearing a dopamine head group were the most active in reducing pro-inflammatory markers among a series of structural analogues, suggesting that the nature of the conjugated amine moiety strongly influences bioactivity of FAAs [26]. In particular, the DHA-derived dopamine (DHDA) has been shown to possess potent anti-inflammatory properties in macrophages as well as in microglial cells [26,27]. DHDA concentration-dependently suppressed the levels of NO, IL-6, MCP-1, MIP3A, and PGE_2_ in RAW264.7 macrophages [27]. The immune-modulating properties of DHDA were also found in microglial cells as it reduced IL-6, MIP3A, and PGE_2_ secreted by LPS-stimulated BV-2 cells [27]. On the other hand, the anti-inflammatory potential of the dopamine conjugate of EPA (*N*-eicosapentaenoyl dopamine, EPDA) has not been studied thus far. The results from the present work show that, at a concentration of 2.5 µM, EPDA decreased NO secreted by LPS-induced RAW264.7 cells. The observed effect is not likely due to EPDA breakdown since its hydrolytic products, EPA and dopamine, did not induce any change in nitrite levels in our experimental set-up [18,27]. NO is a relatively late marker of the inflammatory cascade and by-product of the oxidative deamination of l-arginine, catalyzed by the inducible isoform of nitric oxide synthase (iNOS) [29]. Additionally, we observed that EPDA reduced secreted MCP-1, MIP3A, and IL-6 in a concentration-dependent manner in LPS-induced cells. Remarkably, EPDA was effective in suppressing IL-6 release also at sub-micromolar concentration (0.1 μM). MCP-1 and MIP3A are two chemokines playing important roles in attracting immune cells to the site of inflammation, eventually leading to an increase of the inflammatory reaction [30,31,32]. IL-6 is a cytokine with pleiotropic effects on inflammation, immune response, and cancer. Suppression of IL-6 production has been suggested as a therapeutic strategy for immune-mediated diseases and cancer progression [33,34]. Moreover, we found that EPDA decreased COX-2 gene expression in a concentration-dependent way. This is in line with our previous findings that DHDA and DHA-5-HT inhibited COX-2 at mRNA level in RAW264.7 macrophages [16,27]. On the other hand, DHEA was found to reduce the levels of PGE_2_ and other COX-2-derived eicosanoids without affecting COX-2 at gene expression level [21]. COX-2 represents a potential therapeutic target in many inflammation-associated disorders [35,36,37,38]. In addition, in the present work, we demonstrated that the apparent anti-inflammatory effects found in the murine RAW 264.7 macrophages could be reproduced in a cell line of human origin, as we found that MCP-1 and IL-6 production was decreased upon treatment with EPDA, at the concentration of 1 µM, in LPS-stimulated THP-1 macrophages. This underlines that EPDA possesses a broad spectrum of immune-modulating properties and that the compound is not species-specific. The inhibitory activities elicited by EPDA were regulated at the gene expression level also in human THP-1 macrophages, as we found that mRNA expression of all genes investigated, namely MCP-1, IL-6, COX-2, and IL-1β was reduced by EPDA. IL-1β is a cytokine produced in response to inflammatory stimuli and it is involved in pain, inflammation, and autoimmune conditions. Results from in vivo studies using IL-1β knock-out mice support the major role of IL-1β in modulating nuclear factor (NF)-κB and COX-2 transcription activities induced by systemic inflammation [39,40].

Collectively, EPDA exerts in vitro anti-inflammatory properties in murine and human macrophages. Remarkably, we have previously reported that EPDA displays antitumor activity through autophagy and apoptosis in different breast cancer cell lines, including those exhibiting major aggressiveness and invasiveness [25]. In cancer development, tumor-associated macrophages (TAMs) represent the most abundant cell types in the microenvironment and the major players of the cancer-related inflammation, promoting the acceleration of tumor progression [41]. Remarkably, COX-2 has been shown to promote metastatic potential of breast cancer cells in TAMs [42], while IL-6 signaling is assumed to contribute to a pro-tumor condition by supporting angiogenesis and tumor elusion of immune surveillance [33,34]. Therefore, additional investigations could be of interest to clarify the potential role of EPDA in affecting macrophages within breast tumor microenvironment, to gain new insight into the mechanisms linking inflammation and tumorigenesis. It remains an intriguing question whether EPDA and its congener DHDA also exist in vivo as endogenous metabolites. To our knowledge, this has not yet demonstrated, despite attempts by our group as well as by others [27,43]. This may be due to their complicated bio-analytical behavior, their possibly low abundance, and restriction to discrete sites in the body. Given the lack of knowledge about its endogenous formation, it is well possible that the anti-inflammatory activities of EPDA found in this study represent pharmacological effects and (or) at least effects of the compound at supra-physiological concentrations. At the same time, it is conceivable that EPDA could be formed given the demonstration in vivo of other fatty acid-dopamine conjugates, including *N*-oleoyl dopamine and *N*-arachidonoyl dopamine [44,45] and that of the serotonin conjugates of DHA and EPA [46]. This suggests that the enzymes needed for the synthesis of EPDA could be present in the body. 

## 5. Conclusions

In the present work, we demonstrated the in vitro anti-inflammatory properties of EPDA in both murine and human macrophage models. Further investigations will be devoted to verifying its potential ability in preventing or augmenting the efficacy of more conventional therapeutic approaches for inflammatory disorders and resulting chronic non-communicable diseases.

## Supplementary Materials

The following are available online at https://www.mdpi.com/2072-6643/11/9/2247/s1, Figure S1: The enzymatic synthesis reaction scheme and characterization data of *N*-eicosapentaenoyl dopamine (EPDA), Table S1: Effects of EPDA on LPS-stimulated RAW 264.7 macrophages by XTT / LDH assays, Table S2: Effect of EPDA on viability of LPS-stimulated human THP-1 macrophages.
