# Supplementary material for: N-Eicosapentaenoyl Dopamine, A Conjugate of Dopamine and Eicosapentaenoic Acid (EPA), Exerts Anti-inflammatory Properties in Mouse and Human Macrophages
Source: Nutrients. 2019 Sep 18;11(9):2247. doi: 10.3390/nu11092247 (PMC6769480; doi:10.3390/nu11092247)
Supplement: Supplementary file 1 [file nutrients-11-02247-s001.pdf]

Figure S1. The enzymatic synthesis reaction scheme and characterization data of *N*-eicosapentaenoyl dopamine (EPDA). EPDA was synthesized from EPA and dopamine hydrochloride as described in the main manuscript.

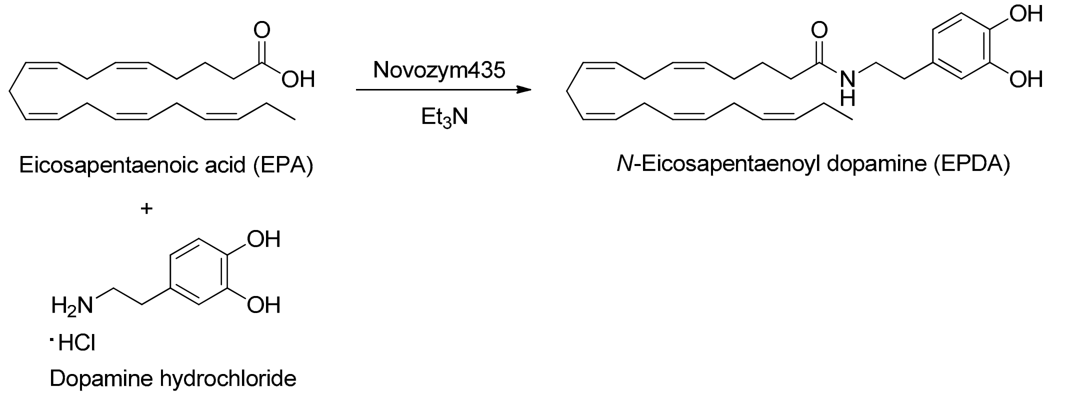

Complete data for characterization of *N*-eicosapentaenoyl dopamine (EPDA):

{(Z,Z,Z,Z,Z)-Eicosa-5,8,11,14,17-pentaenoic acid [2-(3,4-dihydroxyphenyl)ethyl]amide}, colorless oil. IR (film) 3315 (s, br), 3011 (s), 2966 (m), 2930 (w), 1647 (s), 1520 (m) cm^{-1}; ^{1}H NMR δ 6.83-6.50 (m, 3 H, aromatic), 5.82-5.75 (m, 1 H, NH), 5.45-5.25 (m, 10 H, 5 HC=CH), 3.51-3.39 (m, 2 H, $C\underline{H_2}NH$), 2.90-2.72 (m, 8 H, 4 $CH{=}CHC\underline{H_2}CH{=}CH$), 2.67 (t, *J* = 7.1 Hz, 2 H, $C\underline{H_2}CH_2NH$), 2.21-1.99 (m, 6 H, $C\underline{H_2}CH_2C\underline{H_2}C{=}O$ + $C\underline{H_2}CH_3$), 1.74-1.61 (m, 2 H, $CH_2C\underline{H_2}C{=}O$), 0.97 (t, *J* = 7.5 Hz, 3 H, CH_3). (Note: the OH signals were too broad to be detected); ^{13}C NMR δ 174.2, 144.5, 143.3, 132.1, 130.5, 129.0, 128.9, 128.7, 128.4, 128.3, 128.2, 128.1, 127.9, 127.1, 120.5, 115.5, 115.3, 41.1, 36.2, 35.0, 26.6, 25.7, 25.6, 20.6, 14.3; MS m/z 436.2 $[M-H]^-$. MS/MS $[M-H]^-$ (ESI -, 30 eV) m/z 436.2 (56), 134.0 (38), 123.0 (100).

Table S1. Effect of EPDA on LPS-stimulated RAW 264.7 macrophages by XTT / LDH assays

**Treatment**	**Concentration (μM)**	**Viability (%) RAW264.7**	**Cytoxicity (%) RAW264.7**
Vehicle	-	100	100
EPDA	0.01	100 ± 4	116 ± 3
	0.1	105 ± 4	118 ± 12
	1.0	100 ± 5	99 ± 7
	2.5	112 ± 9	109 ± 12
Triton X-100	-	27 ± 2	212 ± 6

Table S2. Effects of EPDA on viability of LPS-stimulated human THP-1 macrophages

**Treatment**	**Concentration (μM)**	**Viability (%) THP-1**		
		24h	48h	72h
Vehicle	-	100	100	100
EPDA	0.1	92 ± 1	104 ± 2	91 ± 8
	1.0	89 ± 6	90 ± 15	88 ± 1
